# Progranulin deficiency promotes neuroinflammation and neuron loss following toxin-induced injury

**DOI:** 10.1172/JCI157161

**Published:** 2022-01-04

**Authors:** Lauren Herl Martens, Jiasheng Zhang, Sami J. Barmada, Ping Zhou, Sherry Kamiya, Binggui Sun, Sang-Won Min, Li Gan, Steven Finkbeiner, Eric J. Huang, Robert V. Farese Jr

Original citation: *J Clin Invest*. 2012;122(11):3955–3959. https://doi.org/10.1172/JCI63113

Citation for this corrigendum: *J Clin Invest*. 2022;132(1):e157161. https://doi.org/10.1172/JCI157161

The sense primer 1 listed for genotyping *GrnKO* was incorrect. The correct sequence is below.

5′-AGTGGGGCTGGCCATCCTC

The authors regret the error.

## Version 1 01/04/2022

Electronic publication

